# Chemical and Stress Resistances of *Clostridium difficile* Spores and Vegetative Cells

**DOI:** 10.3389/fmicb.2016.01698

**Published:** 2016-10-26

**Authors:** Adrianne N. Edwards, Samiha T. Karim, Ricardo A. Pascual, Lina M. Jowhar, Sarah E. Anderson, Shonna M. McBride

**Affiliations:** Emory Antibiotic Resistance Center, Department of Microbiology and Immunology, Emory University School of Medicine, AtlantaGA, USA

**Keywords:** *Clostridium difficile*, *Clostridium difficile* infection (CDI), anaerobe, spores, resistance, germination, sporulation

## Abstract

*Clostridium difficile* is a Gram-positive, sporogenic and anaerobic bacterium that causes a potentially fatal colitis. *C. difficile* enters the body as dormant spores that germinate in the colon to form vegetative cells that secrete toxins and cause the symptoms of infection. During transit through the intestine, some vegetative cells transform into spores, which are more resistant to killing by environmental insults than the vegetative cells. Understanding the inherent resistance properties of the vegetative and spore forms of *C. difficile* is imperative for the development of methods to target and destroy the bacterium. The objective of this study was to define the chemical and environmental resistance properties of *C. difficile* vegetative cells and spores. We examined vegetative cell and spore tolerances of three *C. difficile* strains, including 630Δ*erm*, a 012 ribotype and a derivative of a past epidemic strain; R20291, a 027 ribotype and current epidemic strain; and 5325, a clinical isolate that is a 078 ribotype. All isolates were tested for tolerance to ethanol, oxygen, hydrogen peroxide, butanol, chloroform, heat and sodium hypochlorite (household bleach). Our results indicate that 630Δ*erm* vegetative cells (630 *spo0A*) are more resistant to oxidative stress than those of R20291 (R20291 *spo0A*) and 5325 (5325 *spo0A*). In addition, 5325 *spo0A* vegetative cells exhibited greater resistance to organic solvents. In contrast, 630Δ*erm* spores were more sensitive than R20291 or 5325 spores to butanol. Spores from all three strains exhibited high levels of resistance to ethanol, hydrogen peroxide, chloroform and heat, although R20291 spores were more resistant to temperatures in the range of 60–75°C. Finally, household bleach served as the only chemical reagent tested that consistently reduced *C. difficile* vegetative cells and spores of all tested strains. These findings establish conditions that result in vegetative cell and spore elimination and illustrate the resistance of *C. difficile* to common decontamination methods. These results further demonstrate that the vegetative cells and spores of various *C. difficile* strains have different resistance properties that may impact decontamination of surfaces and hands.

## Introduction

*Clostridium difficile* infection (CDI) is a serious, sometimes fatal, gastrointestinal (GI) disease that has emerged as a major healthcare concern in many hospitals, acute care and long-term care facilities (Rupnik et al., 2009; Dubberke and Olsen, 2012; CDC, 2013; Smits et al., 2016). Susceptibility to CDI can be induced by antibiotic treatment, which disrupts the native intestinal microbiota, creating a niche in which *C. difficile* thrives (Theriot and Young, 2015; Theriot et al., 2016). *C. difficile* spores enter the host by ingestion and germinate into vegetative cells when exposed to bile salts in the GI tract (Sorg and Sonenshein, 2008). *C. difficile* vegetative cells then produce multiple toxins that result in the characteristic diarrhea of CDI (Voth and Ballard, 2005; Shen, 2012; Janoir, 2016). As *C. difficile* vegetative cells transit through the host GI tract, a subset of the cells undergo sporulation, resulting in the formation of dormant spores that are shed in feces, along with vegetative cells (Koenigsknecht et al., 2015). Although CDI is a toxin-mediated disease, the ability for *C. difficile* to form spores is an important virulence factor because the spore serves as the infectious agent and facilitates efficient transmission from host-to-host (Deakin et al., 2012). The physical composition of the spore allows long-term persistence in the environment and confers resistance against desiccation, standard disinfectants and cleaning routines (Driks, 2002, 2003; Ali et al., 2011; Vohra and Poxton, 2011; Dubberke, 2012; Setlow, 2014).

The basic structure of *C. difficile* spores is similar to the spores of *Bacillus subtilis* and other related organisms, with the exception of the exosporium, the outermost layer of the *C. difficile* spore, which is absent in most spore formers (Pizarro-Guajardo et al., 2014, 2016a,b). The spore core, which contains the DNA and other cellular components, is layered by the inner and outer membranes, the cortex and the spore coat (reviewed in Paredes-Sabja et al., 2014). Many orthologs to key proteins that constitute the *B. subtilis* cortex and spore coat are not encoded in *C. difficile* (Henriques and Moran, 2007; Fimlaid et al., 2013), and the receptors and regulatory pathways that govern spore germination are not well conserved (Paredes et al., 2005; de Hoon et al., 2010; Francis et al., 2013; Bhattacharjee et al., 2016), further highlighting the differences between *C. difficile* spores and other bacterial spores. As the individual spore components provide resistance to different stresses (Russell, 1990), from survival in the environment to reactivation within the mammalian gut, elucidating the physical restraints of the *C. difficile* spore is paramount for understanding *C. difficile* biology and developing approaches for the eradication of *C. difficile* in clinical environments.

The purpose of this study was to characterize the resistance properties of the vegetative cells and spores of three *C. difficile* strains, including a derivative of a historical epidemic strain (630Δ*erm*; 012 ribotype), a current epidemic strain (R20291; 027 ribotype) and a strain that is frequently found in animals and humans (5325; 078 ribotype). We observed that each strain has unique resistance properties that likely contribute to their ability to persist as circulating clinical isolates. Our results emphasize the importance of strain variation in *C. difficile* clinical isolates and provide additional evidence for stringent approaches to eradicate *C. difficile* spores and vegetative cells from surfaces.

## Materials and Methods

### Bacterial Strains and Growth Conditions

The bacterial strains used in this study are described in **Table 1**. All *C. difficile* strains were routinely cultured in brain heart infusion-supplemented (BHIS) medium at 37°C in a Coy anaerobic chamber (Bouillaut et al., 2011; Edwards et al., 2013) unless otherwise indicated below.

**Table 1:** Bacterial strains and plasmids.

Plasmid or Strain	Relevant genotype or features	Source, construction or reference
**Strains**		
*Escherichia coli*		
HB101	F^-^ *mcrB mrr hsdS20*(r_B_^-^ m_B_^-^) *recA13 leuB6 ara-14 proA2 lacY1 galK2 xyl-5 mtl-1 rpsL20*	B. Dupuy
MC306	HB101 pRK24 pJS107-*spo0A*178TT	Fimlaid et al., 2013
*Clostridium difficile*		
5325	BAA-1875; ribotype 078	ATCC
630Δ*erm*	Erm^S^ derivative of strain 630; ribotype 012	N. Minton; Hussain et al., 2005
R20291	Clinical isolate; ribotype 027	Stabler et al., 2009
R20291 *spo0A*	R20291 *spo0A*::*erm*	Dawson et al., 2012
MC310 (630 *spo0A*)	630Δ*erm spo0A*::*erm*	Edwards et al., 2014
MC724 (5325 *spo0A*)	5325 *spo0A*::*erm*	This study
**Plasmids**		
pRK24	Tra^+^, Mob^+^; *bla, tet*	Thomas and Smith, 1987
pS107- *spo0A*178TT	Group II intron targeted to *spo0A*	Fimlaid et al., 2013

### Construction of 5325 *spo0A* Mutant

The pJS107-spo0A178TT plasmid containing the group II intron retargeted to the *spo0A* locus was conjugated into strain 5325 (ATCC BAA-1875, ribotype 078; Ho and Ellermeier, 2011; Fimlaid et al., 2013). Thiamphenicol-resistant colonies containing the pJS107-spo0A178TT plasmid were transferred to BHIS plates supplemented with 5 μg ml^-1^ erythromycin. Erythromycin-resistant colonies were isolated and screened for the 2 kb insertion in the *spo0A* locus using primers oMC444 and oMC1122.

### Vegetative Viability Assays

To determine the susceptibility of vegetative cells to a variety of chemical and environmental conditions, the respective *spo0A* mutants of strains 630Δ*erm*, R20291 and 5325 were cultured overnight in BHIS medium. Cultures were grown to mid-exponential phase (OD_600_ ∼0.5) and diluted 1:10 into fresh BHIS. When the cultures reached an OD_600_ ∼1.0, cells were exposed to various concentrations of chemicals for 15 min as follows: (a) 1 ml culture aliquots were mixed with 5–100 μl butanol (Fisher Scientific), chloroform (Acros Organics), hydrogen peroxide (3% H_2_O_2_ as a commercially available aqueous solution) or sodium hypochlorite (8.25% NaOCl as commercially available concentrated household bleach) to achieve the indicated final concentrations, (b) 6 ml culture aliquots were placed in 15 ml screw-cap polypropylene conical tubes, cells were pelleted in a benchtop centrifuge at 4,000 rpm for 5 min, suspended in equal volume pre-reduced 1X PBS, divided into 1 ml aliquots and mixed with 5–100 μl concentration of NaOCl as indicated above, (c) 500 μl of culture was combined with 100–300 μl 95% ethanol and brought to a final volume of 1 ml with dH_2_O, or (d) 1 ml of culture was placed in 8 ml glass screw-cap tubes and incubated at the indicated temperature in a heated water bath for 20 min. pH was measured using a benchtop Accumet AB150 pH/MV meter. For exposure to oxygen, 2 ml culture was placed in a sterile petri dish, removed from the anaerobic chamber, sealed with parafilm to reduce evaporation and aliquots were removed after 30 min, 1, 3, 6, 9, 12, and 24 h incubation. After exposure, cells were serially diluted in either pre-reduced BHIS medium or 1X PBS and plated onto BHIS supplemented with 0.1% taurocholate. There was no difference in colony forming units (CFU) recovered from either diluent (data not shown). Untreated controls for each culture were diluted and plated to BHIS containing taurocholate prior to treatment to enumerate the initial CFU. Plates were enumerated after at least 24 h incubation at 37°C.

### Preparation of Spores

Isolated spores were prepared as previously described Edwards et al. (2013) with the following alterations. Briefly, overnight cultures of *C. difficile* strains grown in BHIS were diluted into fresh BHIS and grown to mid-exponential phase (OD_600_ ∼0.5). Two hundred microliter culture was spread onto 70:30 sporulation agar (Fimlaid et al., 2013) and incubated anaerobically at 37°C for ∼48 h. The plates were removed from the anaerobic chamber and exposed to oxygen at room temperature for 24 h. Cells were scraped from plates, suspended in 1X PBS and washed once. Spore suspensions were placed in flasks and exposed to oxygen at room temperature for 7–10 days to kill all vegetative cells. The surviving spores were subsequently rinsed twice with 1X PBS. The final spore stocks were enumerated on BHIS supplemented with 0.1% taurocholate and stored at a final concentration of either 1 × 10^7^ CFU ml^-1^ in 1X PBS supplemented with 1% BSA to prevent clumping of spores or 1 × 10^8^ CFU ml^-1^ in 1X PBS. BSA was omitted from the higher concentration spore stocks due to precipitation upon addition of some chemicals; however, the presence or absence of BSA did not affect the survival rates of spores in any tested condition. These spore stocks are stable at room temperature for at least a month (data not shown).

### Spore Resistance Assays

Hundred microliter aliquots of 1 × 10^8^ CFU ml^-1^ spores were incubated in the indicated concentration of chemicals for 15 min to ensure a final concentration of 1 × 10^7^ CFU ml^-1^ spores, which is a concentration commonly used to test spore properties (Lawley et al., 2009). To test spore survival in a range of temperatures, 1 ml 1 × 10^7^ CFU ml^-1^ spores were placed in 8 ml glass screw-cap tubes and incubated at the indicated temperature in a heated water bath for 20 min. To test the efficacy of NaOCl in BHIS, 0.6 ml spore aliquots in 1X PBS were pelleted at 15000 rpm for 15 min, and 0.55 ml of supernatant was removed to minimize disruption of the spore pellet. Spores were subsequently suspended in equal volume BHIS and treated as described above. At the indicated times, serial dilutions were performed in 1X PBS and plated onto BHIS plates supplemented with 0.1% taurocholate. Colonies were enumerated from plates after a minimum of 36 h incubation.

### Phase Contrast Microscopy

Phase contrast microscopy was performed as previously described (Edwards et al., 2014). Briefly, cultures were pelleted or cells were scraped off plates, suspended in 500 μl BHIS and pelleted. The supernatant was decanted, and 2 μl of cell suspension was placed on a prepared slide containing a thin 0.7% agarose pad. Phase contrast microscopy was performed using a X100 Ph3 oil immersion objection on a Nikon Eclipse Ci-L microscope while images were captured using a DS-Fi2 camera.

### Statistical Analyses

To evaluate the statistical significance, a two-way analysis of variance (ANOVA), followed by a Dunnett’s multiple comparisons test, was used to compare the control to the concentration, length of time or temperature of the indicated conditions. To determine the significance of the germination frequency of purified spores, a one-way ANOVA was performed, followed by a Tukey’s multiple comparisons test. *P* ≤ 0.05 was considered statistically significant, and all statistical analyses were performed using Microsoft Excel or GraphPad Prism 6.

### Accession Numbers

The accession numbers for strains used in this study are as follows: 630 (GenBank accession no. AM180355); R20291 (GenBank accession no. FN545816); M120 (078 reference strain; GenBank accession no. FN665653).

## Results

### Construction of a *spo0A* Mutant in 5325 (078 Ribotype)

Because *C. difficile* is shed from the host as both vegetative cells and spores (Wilson et al., 1982; Koenigsknecht et al., 2015; Edwards et al., 2016), we first examined the sensitivities of vegetative cells to environmental stresses. *C. difficile* heterogeneously and asynchronously sporulates (Fimlaid et al., 2013). Thus, to ensure no spores were present in vegetative cell assays, we studied *C. difficile* mutants that are unable to form spores. The transcriptional regulator, Spo0A, serves as the master regulator of sporulation in all studied endospore-forming bacteria. Spo0A is required for the formation of spores and transmission of *C. difficile* (Deakin et al., 2012). To ensure all cells within a studied population were vegetative, we utilized a 630Δ*erm spo0A*::*erm* mutant (hereafter referred to as 630 *spo0A*; MC310; 012 ribotype) and a R20291 *spo0A*::*erm* mutant (R20291 *spo0A*; 027 ribotype) (Dawson et al., 2012; Edwards et al., 2014). In addition, we created the same insertional mutation in the *spo0A* locus in 5325 (5325 *spo0A*; MC724; 078 ribotype). The insertion of a retargeted group II intron in the 5325 *spo0A* gene was confirmed by PCR amplification (**Table 2**; Supplementary Figure S1A). Further, phase contrast microscopy of the 5325 *spo0A* mutant revealed the absence of phase bright spores, indicating that the *spo0A* gene product was inactivated (Supplementary Figure S1B), matching the phenotype obtained in a previously published *spo0A* mutant in a 078 ribotype strain (Mackin et al., 2013).

**Table 2:** Oligonucleotides.

Primer	Sequence (5′→3′)	Use/locus tag/reference
oMC444	5′-GGAATACACAGGAGGTATCGTACA-3′	Confirmation of 5325 *spo0A* mutant
oMC819	5′-GTGCGGCTGGATCACCTCCT-3′	PCR ribotyping (Bidet et al., 2000)
oMC820	5′-CCCTGCACCCTTAATAACTTGACC-3′	PCR ribotyping (Bidet et al., 2000)
oMC1122	5′-AACCCTACTGGTTATACCGTTTCG-3′	Confirmation of 5325 *spo0A* mutant

### *C. difficile* Vegetative Cell and Spore Resistance to Ethanol

**FIGURE 1:**
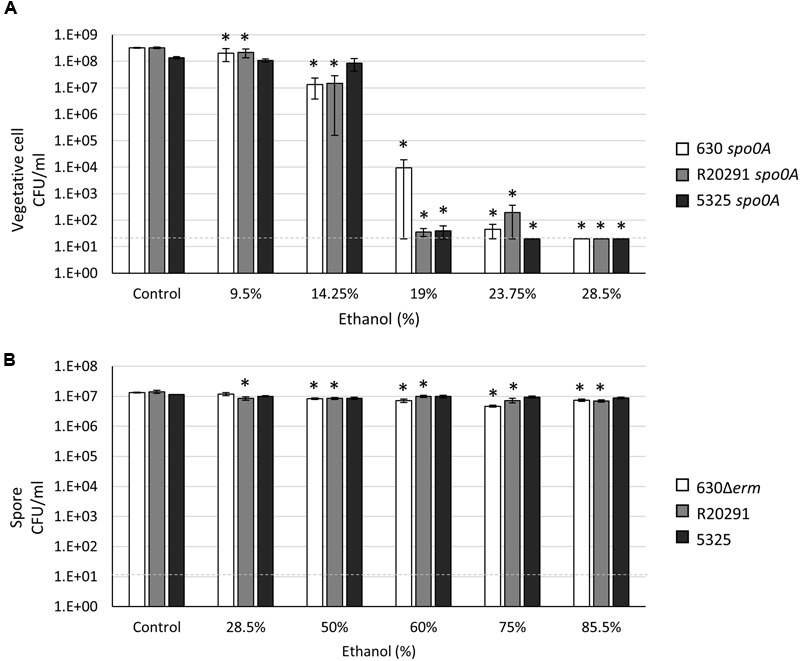
***Clostridium difficile* vegetative cells are sensitive to ethanol, while spores are highly resistant to ethanol. (A)** Survival of 630 *spo0A*, R20291 *spo0A* and 5325 *spo0A* vegetative cells grown to an OD_600_ ∼1.0 in BHIS medium and **(B)** 630Δ*erm*, R20291 and 5325 spores in 1X PBS after exposure to the indicated concentrations (v/v) of ethanol for 15 min. The means and standard error of the means for at least three biological replicates are shown; the limit of detection equals 20 CFU ml^-1^ for vegetative cells and 10 CFU ml^-1^ for spores and is denoted by a dashed line. Asterisks indicate a *P* value of <0.05 as determined by two-way ANOVA followed by a Dunnett’s multiple comparisons test to compare the condition to the corresponding control of individual strains.

Alcohol-based hand sanitizers containing 60–80% ethanol or isopropanol have emerged as a primary line of defense in preventing the transmission of infectious agents in healthcare settings. However, *C. difficile* spores are recalcitrant to alcohol exposure and are not removed from hands after use of alcohol rubs (Oughton et al., 2009; Edmonds et al., 2012; Nerandzic et al., 2015). To compare the resilience of *C. difficile* spores and vegetative cells to ethanol exposure, we first performed vegetative viability assays with the *spo0A* mutants in various concentrations of ethanol for 15 min. Both the 630 *spo0A* and R20291 *spo0A* numbers were reduced by ∼95% in 14.25% ethanol, whereas survival of the 5325 *spo0A* mutant was marginally affected at this concentration (∼35% reduction; **Figure 1A**). However, the vegetative cells of the three ribotypes tested were consistently reduced to undetectable levels in 28.5% ethanol, indicating that prolonged exposure to relatively high concentrations of alcohol can kill all *C. difficile* vegetative cells.

Depending on the methods used to isolate and purify spores, we have observed that brief ethanol or heat exposure (15–30 min) can enhance spore germination (data not shown). Based on these observations, we chose to isolate *C. difficile* spores following long-term exposure to oxygen, as described in “Materials and Methods,” rather than by ethanol and/or heat exposure. This method reproducibly produced stocks with phase bright, viable spores containing no viable vegetative cells and some cell debris (Supplementary Figure S2). As previously demonstrated, *C. difficile* spores germinate efficiently in the presence of the bile salt, taurocholate, and the amino acid, glycine (Sorg and Sonenshein, 2008). Remarkably, we observed that regardless of the method used to eliminate vegetative cells, the 5325 (078 ribotype) spores consistently germinated and outgrew in medium lacking taurocholate at a much higher frequency (∼75-fold increase) than either the 630Δ*erm* (012) or R20291 (027) spores (**Table 3**). This observation suggests that 5325 spores germinate using a taurocholate-independent mechanism, or more likely, that this strain has an increased ability to scavenge taurocholate from medium with components from animal by-products [i.e., brain-heart infusion (BHI) medium; see Discussion].

**Table 3:** Germination frequency of *C. difficile* 012, 027, and 078 spores in the absence and presence of taurocholate.

Strain	Ribotype	Taurocholate-independent germination frequency^a^
630Δ*erm*	012	2.04 × 10^-4^ ± 9.36 × 10^-5^
R20291	027	2.50 × 10^-4^ ± 2.24 × 10^-4^
5325	078	**1.72 × 10**^-^**^2^ ± 9.05 × 10**^-^**^5^**

*^a^Mean germination frequency is calculated from two independently prepared spore stocks as CFU ml^-*1*^ enumerated on BHIS divided by CFU ml^-*1*^ enumerated on BHIS supplemented with 0.1% taurocholate (total spores). Bold indicates *P* < 0.05 as determined by a one-way ANOVA followed by Tukey’s multiple comparisons test.*

To measure *C. difficile* spore resistance to ethanol, concentrated spore stocks suspended in 1X PBS were mixed to the indicated final concentration of ethanol and incubated for 15 min. The final concentration of spores in these assays was 1 × 10^7^, which mimics the concentration used in a previous spore resistance study (Lawley et al., 2009) and closely reflects the number of CFUs enumerated from hamster feces during infection (Edwards et al., 2014, 2016). As previously reported (Lawley et al., 2009), *C. difficile* spores were extremely resistant to high levels of ethanol, with all strains exhibiting high survival rates (≥50%) even in concentrations of ethanol as high as 85.5% (**Figure 1B**). Notably, the 5325 spores had higher survival rates in higher concentrations of ethanol than did the 630Δ*erm* or R20291 spores, although this effect was not statistically significant (78% spore survival vs. 56% and 50%, respectively, in 85.5% ethanol; Supplementary Figure S3). Altogether, these results indicate that *C. difficile* spore exposure to ethanol does not inactivate spores.

### *C. difficile* Resistance to Additional Organic Solvents

**FIGURE 2:**
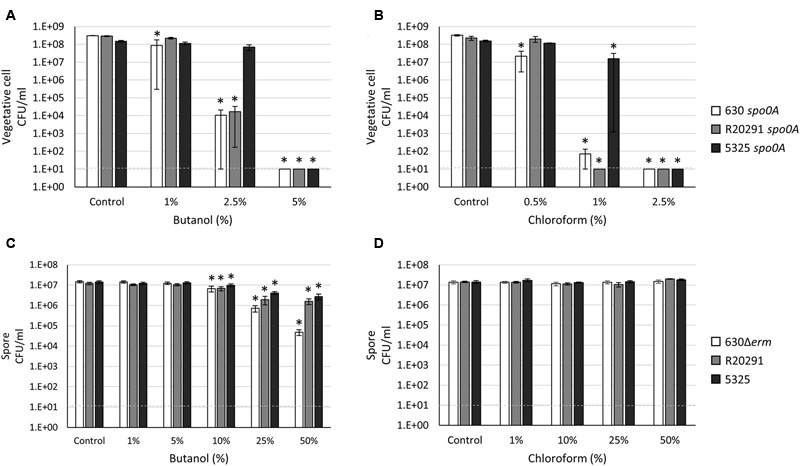
***Clostridium difficile* 5325 vegetative cells exhibit greater resistance to solvents while R20291 spores are more sensitive to butanol. (A,C)** Survival of 630 *spo0A*, R20291 *spo0A* and 5325 *spo0A* vegetative cells grown to an OD_600_ ∼1.0 in BHIS medium after a 15 min exposure to the indicated concentrations (v/v) of **(A)** butanol and **(C)** chloroform. **(B,D)** Survival of 630Δ*erm*, R20291 and 5325 spores in 1X PBS after a 15 min exposure to **(B)** butanol and **(D)** chloroform. The means and standard error of the means for at least three biological replicates are shown; the limit of detection is 10 CFU ml^-1^ and is denoted by a dashed line. Asterisks indicate a *P*-value of <0.05 as determined by two-way ANOVA followed by a Dunnett’s multiple comparisons test to compare the condition to the corresponding control of individual strains.

*Clostridium* species and related organisms carry out solventogenesis, a process during which growth slows and solvents such as acetone, butanol, and ethanol are produced. For this reason, *Clostridium* and related organisms are used for the production of biofuels, including butanol, which generally inhibit microbial growth. As such, a frequent limiter for solvent production is the high level of solvent toxicity exhibited in relatively low concentrations (Ezeji et al., 2010). As solvent toxicity is understudied in *C. difficile*, we tested the susceptibility of *C. difficile* vegetative cells and spores to butanol as described in “Materials and Methods.” Surprisingly, a significant number of the 5325 *spo0A* cells survived in 2.5% butanol compared to the 630 *spo0A* and R20291 *spo0A* strains (**Figure 2A**; 50% survival versus 0.003 and 0.01% survival, respectively). While long-term viability and the ability to grow uninhibited in relatively high concentrations of butanol were not tested, these data suggest that the 5325 strain has the ability to withstand greater concentrations of butanol compared to other common *C. difficile* isolates. Spores of all three *C. difficile* strains demonstrated some sensitivity to increasing concentrations of butanol (**Figure 2B**). However, when spores were incubated with 50% butanol, the 630Δ*erm C. difficile* spore CFU were reduced to <1%, compared to ∼13% and 19% survival rates for the R20291 and 5325 spore populations, respectively, suggesting that the 630Δ*erm* spores are more susceptible to butanol.

We also measured the sensitivity of *C. difficile* vegetative cells and spores to chloroform, an organic solvent sometimes used in sporulation assays for *Bacillus subtilis* and other clostridial organisms (Milhaud and Balassa, 1973; Schiott and Hederstedt, 2000; McBride and Haldenwang, 2004; Tracy et al., 2011). The 5325 *spo0A* mutant demonstrated greater resistance to chloroform compared to the 630 *spo0A* and R20291 *spo0A* mutants, with 10% of the 5325 *spo0A* population surviving 1% chloroform exposure (**Figure 2C**), indicating that the 5325 vegetative cells are able to tolerate greater concentrations of chloroform. *C. difficile* spores were unaffected by chloroform exposure, but incubation in chloroform resulted in a small, but not statistically significant, increase in CFU in all strains (**Figure 2D**). However, this effect was not as consistent in higher concentrations of chloroform.

### *C. difficile* Resistance to Oxidative Stress

**FIGURE 3:**
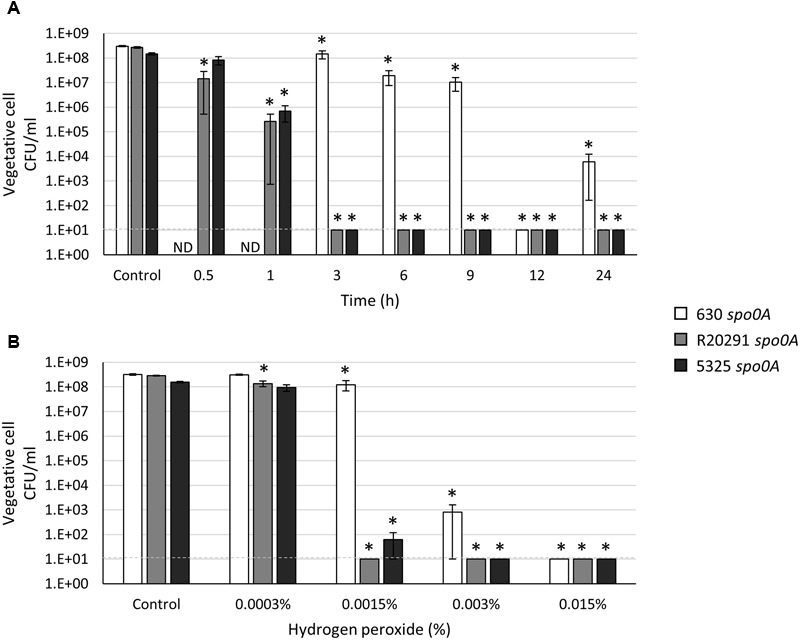
***Clostridium difficile* 630 *spo0A* vegetative cells demonstrate greater resistance to oxidative stress.** Survival of 630 *spo0A*, R20291 *spo0A* and 5325 *spo0A* vegetative cells grown to an OD_600_ ∼1.0 in BHIS medium after exposure to **(A)** atmospheric oxygen after the indicated length of time (h) or **(B)** hydrogen peroxide (H_2_O_2_) for 15 min. The means and standard error of the means for at least three biological replicates are shown; the limit of detection is 10 CFU ml^-1^ and is denoted by a dashed line. Asterisks indicate a *P*-value of <0.05 as determined by two-way ANOVA followed by a Dunnett’s multiple comparisons test to compare the condition to the corresponding control of individual strains. ND, not determined.

*Clostridium difficile* vegetative cells are strict anaerobes and are known to be sensitive to low levels of oxygen (Holy and Chmelar, 2012). However, previous results in our lab demonstrated that some *C. difficile* vegetative cells present in hamster fecal samples survive at least 24 h in fecal pellets suspended in 1X PBS and stored aerobically (Edwards et al., 2016). These data led us to hypothesize that oxygen tolerance may be exhibited by *C. difficile* vegetative cells for short, but significant, periods of time. Although the transmission of non-sporulating *C. difficile* is almost eliminated in the mouse model of *C. difficile* infection (CDI) (Deakin et al., 2012), significant numbers of vegetative cells are shed and recovered in the stool (Koenigsknecht et al., 2015). It is unknown if vegetative cells play a role in transmission in clinical settings. To determine the extent of *C. difficile* vegetative cell oxygen tolerance, the *spo0A* mutant strains were grown to an OD_600_ of 1.0, plated for viability anaerobically and aliquots of the cultures were exposed to atmospheric oxygen for the indicated amount of time, serially diluted and plated anaerobically to enumerate surviving cells. As shown in **Figure 3A**, the 630 *spo0A* mutant strain was significantly more tolerant to atmospheric oxygen exposure than either the R20291 *spo0A* or 5325 *spo0A* mutant strains. R20291 *spo0A* and 5325 *spo0A* survival dropped precipitously after 1 h exposure to oxygen, and no surviving cells were recovered after 3 h oxygen exposure. These results demonstrate that the 630Δ*erm* strain is able to survive brief oxygen exposure, perhaps providing additional opportunity for spread of the pathogen.

We next determined the effects of an important source of oxidative stress, the common antiseptic and oxidative burst component, hydrogen peroxide (Fawley et al., 2007; Slauch, 2011). Previous studies genetically linked the abilities for *Clostridium acetobutylicum*, a related anaerobe, to tolerate both oxygen and hydrogen peroxide exposures (Hillmann et al., 2008, 2009; Riebe et al., 2009). We asked whether *C. difficile* survival in oxygen correlates with hydrogen peroxide tolerance, as is observed with *C. acetobutylicum*. Spores from all three strains were resistant to up to 1.5% hydrogen peroxide (Supplementary Figure S4), confirming previous results performed with similar or unknown concentrations of hydrogen peroxide (Fawley et al., 2007; Lawley et al., 2010). In contrast, the vegetative cells of the 630 *spo0A* mutant exhibited increased resistance to hydrogen peroxide exposure compared to the R20291 *spo0A* and 5325 *spo0A* mutants (**Figure 3B**), similar to the atmospheric oxygen survival trials. These data suggest that the increased vegetative cell oxygen and hydrogen peroxide tolerance exhibited by the 630Δ*erm* strain may be correlative, as in *C. acetobutylicum*.

### *C. difficile* Spores Tolerate High “Wet Heat” Temperatures for a Short Period of Time

**FIGURE 4:**
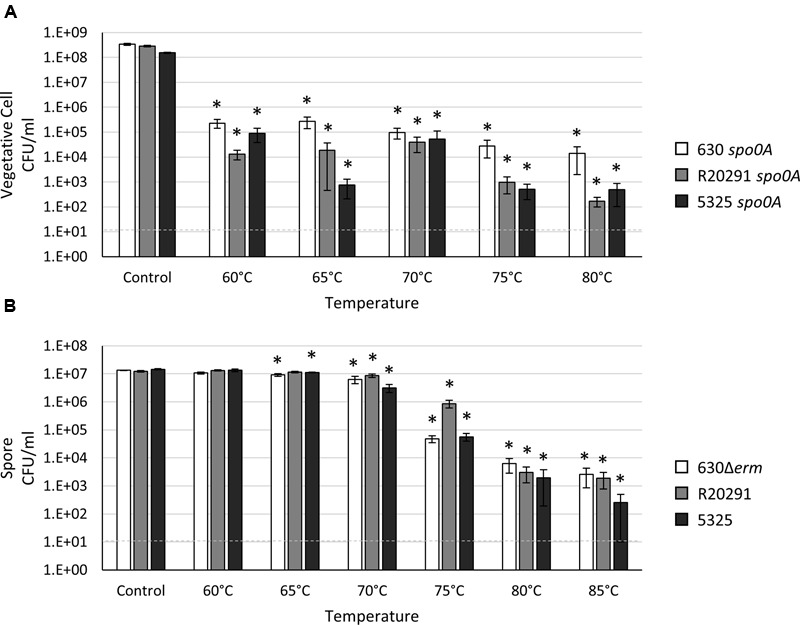
***Clostridium difficile* spores are resistant to high temperatures.** Survival of **(A)** 630 *spo0A*, R20291 *spo0A* and 5325 *spo0A* vegetative cells grown to an OD_600_ ∼1.0 in BHIS medium or **(B)** 630Δ*erm*, R20291 and 5325 spores in 1X PBS after a 20 min exposure to the indicated temperature. The means and standard error of the means for at least three biological replicates are shown; the limit of detection is 10 CFU ml^-1^ and is denoted by a dashed line. Asterisks indicate a *P*-value of <0.05 as determined by two-way ANOVA followed by a Dunnett’s multiple comparisons test to compare the condition to the corresponding control of individual strains.

One advantage to bacterial spore formation is protection from extreme temperature variations. Wet heat resistance is the ability for spores to survive high temperatures when suspended in an aqueous solution (Setlow, 2006). This property is a universal characteristic of bacterial spores, but differences in heat tolerance between *Bacillus* and other spore-forming species have been noted (Murrell and Scott, 1966; Setlow, 2014). While *B. subtilis* spores can easily survive exposure to 80°C (Milhaud and Balassa, 1973; Setlow, 2006; Leggett et al., 2012), previous studies in *C. difficile* have demonstrated that *C. difficile* spores are comparatively wet heat-labile (Lawley et al., 2009). *C. difficile* spores have been reported to withstand long-term exposure (>24 h) to 60°C and up to 3 h at 70°C (Lawley et al., 2009), and wet heat-resistance assays are often used to select for *C. difficile* spores in a heterogenous population (Burns et al., 2011; Paredes-Sabja and Sarker, 2011; Fimlaid et al., 2015). We characterized the ability of *C. difficile* vegetative cells and spores to survive exposure to a range of wet heat temperatures for 20 min. The number of *C. difficile* vegetative cells recovered after exposure to 60°C or greater temperature resulted in a ∼3-log loss for all three strains; however, we variably recovered some viable vegetative cells following incubation in 80°C (**Figure 4A**). Exposure to 60°C resulted in a slight, but statistically insignificant, loss of spore viability for all strains (**Figure 4B**; Supplementary Figure S5). The loss of 630Δ*erm* and 5325 spore viability was more pronounced from 65 to 75°C compared to R20291 spores, while all strains exhibited a significant decrease in spore recovery at 80°C (**Figure 4B**; Supplementary Figure S5). The R20291 spores exhibited a modest, but consistent, advantage in heat tolerance compared to the 630Δ*erm* and 5325 spores (**Figure 4B**; Supplementary Figure S5). Viable spores were detectable from all three strains after exposure to 85°C, although the spore load was reduced by at least ∼4-logs, comparable to previous observations (Rodriguez-Palacios and Lejeune, 2011). These data indicate that a substantial proportion of *C. difficile* spores are susceptible to wet heat-killing at temperatures below 85°C, but heat susceptibility is strain-dependent. These results should be taken into consideration when performing heat-resistance assays to quantitate sporulation within a population. Unsurprisingly, temperatures greater than 85°C are required to completely eliminate all *C. difficile* spores when in an aqueous environment.

### Household Bleach Is Effective in Eliminating Both *C. difficile* Vegetative Cells and Spores at an Alkaline pH but Loses Efficacy at a Neutral pH

**FIGURE 5:**
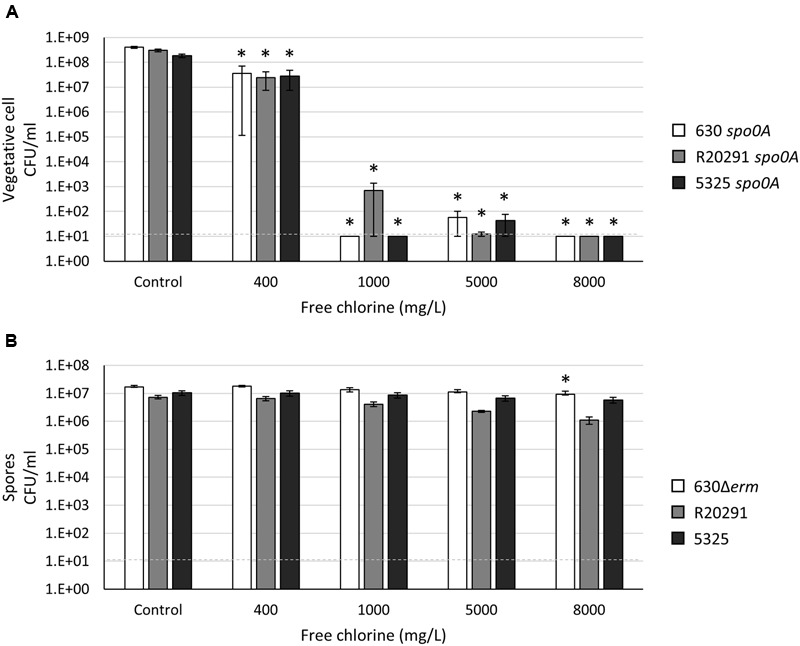
***Clostridium difficile* vegetative cells and spores are resistant to sodium hypochlorite (household bleach) at a physiological pH.** Survival of **(A)** 630 *spo0A*, R20291 *spo0A* and 5325 *spo0A* vegetative cells grown to an OD_600_ ∼1.0 in BHIS medium or **(B)** 630Δ*erm*, R20291 and 5325 spores suspended in BHIS medium after exposure to 400 mg l^-1^, 1000 mg l^-1^, 5000 mg l^-1^, 8000 mg l^-1^ free chlorine (FC) for 15 min. The means and standard error of the means for at least three biological replicates are shown; the limit of detection is 10 CFU ml^-1^ and is denoted by a dashed line. Asterisks indicate a *P*-value of <0.05 as determined by two-way ANOVA followed by a Dunnett’s multiple comparisons test to compare the condition to the corresponding control of individual strains.

Sodium hypochlorite (NaOCl), the active chemical present in household bleach, is one of the most effective and commonly recommended agents for general disinfection (McDonald et al., 2012). Household bleach is commercially available as an aqueous solution of 5.25–8.25% NaOCl, which correlates to a standard concentration of approximately 5000–8000 mg l^-1^ free chlorine [FC or parts per million (ppm)]. Previous studies have demonstrated that acidified and household bleach significantly reduce or completely eliminate the number of *C. difficile* spores on hard surfaces (Perez et al., 2005; Alfa et al., 2010; Omidbakhsh, 2010). However, we asked whether household bleach was effective in eliminating a high concentration of vegetative cells and spores in an aqueous suspension, as may be the case in some patient care situations. To first assess the microbicidal capacity of household bleach against *C. difficile* vegetative cells in suspension, we added household bleach at 400 mg l^-1^, 1000 mg l^-1^, 5000 mg l^-1^, and 8000 mg l^-1^ FC (equivalent to 0.5, 1.25, 6.25, and 10% bleach solutions) to the asporogenous population of the three *C. difficile* strains. As before, these strains were grown in the standard medium for cultivation, BHIS, pH 6.8. At an OD_600_ of 1.0, the pH of the culture was 5.98 ± 0.04. When bleach was added to the culture, the pH was increased in a dose-dependent manner, favoring hypochlorite (ClO^-^) formation [final culture pH = 6.04 ± 0.04 in 400 mg l^-1^ (0.5%) household bleach; final culture pH = 6.60 ± 0.02 in 5000 mg l^-1^ (6.25%) household bleach.] After a 15 min incubation in 400 mg l^-1^ (0.5%) household bleach, the vegetative cell population of all three strains was decreased by ∼85–90% (**Figure 5A**). Few vegetative cells were recoverable after incubation in 1000 mg l^-1^ (1.25%) and 5000 mg l^-1^ (6.25%) household bleach, and viable *C. difficile* cells were reduced to below the limit of detection (<10 CFU ml^-1^) in 8000 mg l^-1^ (10%) household bleach (**Figure 5A**). As pH alters the ratio of hypochlorite (HOCl) to the hypochlorite ion (OCl^-^), and thus, the effectiveness of bleach (Baumann and Ludwig, 1962), these data suggested that the pH of the aqueous solution may alter the effectiveness of sodium hypochlorite. To test this hypothesis, vegetative cells grown to an OD_600_ of 1.0 in BHIS medium were pelleted and suspended in pre-reduced 1X PBS (pH 7.4). The addition of household bleach to 1X PBS resulted in an alkaline suspension (pH 8.4–10, depending on the final concentration of bleach). A 15 min incubation in household bleach at all concentrations reduced the number of recoverable viable vegetative cells to below the limit of detection (<10 CFU ml^-1^; data not shown).

We next determined the efficacy of household bleach against *C. difficile* spores suspended in either 1X PBS or BHIS medium. As with *C. difficile* vegetative cells suspended in 1X PBS, a 15 min exposure to all concentrations of bleach was effective in reducing viable spores to below the limit of detection (<10 CFU ml^-1^; data not shown). Surprisingly, *C. difficile* 630Δ*erm*, R20291 and 5325 spores suspended in BHIS were able to withstand a 15 min exposure to household bleach, even at the highest concentration of 8000 mg l^-1^ (10%) household bleach (**Figure 5B**). These data suggest that the pH of liquid biohazard spills (e.g., feces) may alter the effectiveness of sodium hypochlorite. As a result, higher concentrations of bleach and/or the use of additional components to ensure an alkaline pH may be necessary to completely eliminate *C. difficile* vegetative cells and spores in biohazard spills.

## Discussion

*Clostridium difficile* infections have quickly emerged as a significant healthcare burden, and result in billions of dollars per year in additional healthcare-related expenses (Kwon et al., 2015; Levy et al., 2015). The environmental reduction of *C. difficile* spores is a major concern in the prevention of efficient transmission in hospitals and acute and long-term care facilities. While sodium hypochlorite (household bleach) has traditionally been recommended for sanitization in these settings, studies have shown that without frequent and thorough cleaning, detection and transmission of *C. difficile* can occur (Macleod-Glover and Sadowski, 2010; Lessa et al., 2012). To better understand how the resistance properties of *C. difficile* could impact survival and transmission, this study investigated the persistence of vegetative cells and spores after exposure of different *C. difficile* strains to a variety of chemicals and temperatures.

As expected, we observed greater effects for all of the chemicals and conditions tested on the viability of vegetative cells, compared to that of spores. The highest concentrations of ethanol (28.5%), butanol (5%), chloroform (2.5%), hydrogen peroxide (0.015%) or bleach (8000 mg l^-1^) tested were sufficient to decrease the vegetative cell counts for all strains to below detectable limits (∼10 CFU/ml). But, some treatments were more effective against vegetative cells of some strains than others. In particular, the 630 *spo0A* strain demonstrated higher resistance to oxygen exposure and hydrogen peroxide than the R20291 *spo0A* and 5325 *spo0A* strains examined. Conversely, the 5325 *spo0A* strain displayed greater resistance to killing by the organic compounds butanol and chloroform than the 630 *spo0A* and R20291 *spo0A* strains. These results reveal strain-dependent differences in survival that are specific to actively growing cells and independent of spore structure. Because *C. difficile spo0A* mutants exhibit pleiotropic phenotypes, we tested the 630 *spo0A* isogenic parent, 630Δ*erm*, in a subset of conditions, and found no difference in survival rates (Supplementary Figure S6), indicating that the *spo0A* mutant strain behaves similarly to the parent strain.

To our knowledge, aerotolerance of *C. difficile* vegetative cells has not been mechanistically studied. The observation that oxygen sensitivity and hydrogen peroxide resistance are strain-dependent properties may provide insight into the mechanisms by which *C. difficile* tolerates low levels of oxidative stress. A mutation in the oxidative stress repressor (*perR*) in the related organism, *Clostridium acetobutylicum*, confers prolonged aerotolerance and higher resistance to hydrogen peroxide (H_2_O_2_; Hillmann et al., 2008). A single genetic pathway in *C. acetobutylicum*, which includes PerR, is responsible for detoxifying both atmospheric oxygen and reactive oxygen species (ROS), including those produced by hydrogen peroxide (Riebe et al., 2009). Although *C. acetobutylicum* is more aerotolerant than *C. difficile*, the fact that resistance to oxygen exposure and ROS correlates with individual strains suggests that *C. difficile* may also utilize a single genetic pathway to combat multiple sources of oxidative stress. The infectious dose of *C. difficile* is unknown, but is presumed to be extremely low (<10), as is observed for the hamster model of acute CDI (Larson et al., 1978). Consequently, it is possible that vegetative cells play a minor role in the spread of *C. difficile* if vegetative cells from feces can survive short periods of exposure to atmospheric oxygen.

As anticipated, spores of all three strains were inherently resistant to many of the chemicals tested, with ethanol, chloroform and hydrogen peroxide proving ineffective at any practical concentration that could be assessed. Strain-dependent differences were observed for spore resistance to butanol, which was more effective against strain 630Δ*erm*, and wet heat, which more readily killed 630Δ*erm* and 5325. The highest wet-heat temperature examined, 85°C, was not sufficient to completely destroy all spores for any of the strains examined.

As environmental control is one of the critical measures in preventing the spread of CDI, the use of the most effective cleaning products, along with adequate cleaning procedures (Eckstein et al., 2007), is necessary to reduce the environmental sources of *C. difficile* spores (Macleod-Glover and Sadowski, 2010). Our results confirm that spores are recalcitrant to high concentrations of ethanol, the primary active ingredient in alcohol-based hand sanitizers, and relatively high concentrations of hydrogen peroxide (<3%), which is an active ingredient in several hospital cleaning agents. A previous study demonstrated that 1% hydrogen peroxide can reduce spore viability by ∼75% when incubated with 10^6^ spores/ml. We observed ∼25–40% reduction that was not statistically significant (Supplementary Figure S4), and we used a higher concentration of spores in our tests (10^6^ vs. 10^7^ spore CFU/ml) (Lawley et al., 2010). Further, Lawley et al. (2010) found that exposure to a 10% hydrogen peroxide solution reduced viable spores to below their limit of detection (<2 CFU ml^-1^). Some studies have shown that the use of hydrogen peroxide-based hospital cleaning agents [e.g., G-force, accelerated hydrogen peroxide (AHP) and stabilized hydrogen peroxide (SHP)] in clinical settings can reduce, but not completely eliminate, detection of *C. difficile* or patient cases of CDI (Alfa et al., 2010; Horn and Otter, 2015; Steindl et al., 2015). Because spores are highly resistant to these compounds in the laboratory and strong, stable solutions of hydrogen peroxide are necessary for spore inactivation, our results strongly suggest that these products not be used for decontamination, and confirm that a strong dilution of household bleach at an alkaline pH is the best choice for environmental control of *C. difficile* (Wilcox et al., 2003; Eckstein et al., 2007).

We observed that spores of the 5325 strain appeared to germinate in a taurocholate-independent manner on BHIS plates at a significantly higher frequency than 630Δ*erm* or R20291 spores. Other *C. difficile* clinical isolates have exhibited germination in the absence of bile salts, and no correlation between ribotypes was noted (Heeg et al., 2012). However, these spores were heat treated at 60°C for 25 min before plating, which may increase the germination frequency (Rodriguez-Palacios and Lejeune, 2011). A recent study revealed that all tested *C. difficile* strains, including those in the previous study that germinated and resumed growth in medium lacking taurocholate, required taurocholate and glycine to germinate *in vitro*, with the exception of CD 2351, a 078 clinical isolate, which did not require glycine (Bhattacharjee et al., 2016). Further, some spores exhibited a significantly increased affinity to taurocholate (Bhattacharjee et al., 2016), suggesting that spores from these strains require lower concentrations of taurocholate to activate germination. Thus, the difference in frequencies of taurocholate-independent germination by 5325 compared to 630Δ*erm* and R20291 may result from an increased ability for 5325 to bind low levels of taurocholate. The variation in affinity to bile salts of various *C. difficile* clinical isolates may reflect the availability of nutrients and ratio of bile salts present in the primary host GI tract.

This study further highlights the broad variations observed in *C. difficile* clinical and animal isolates in regards to many physiological processes, including sporulation, germination and toxin production (Burns et al., 2010; Heeg et al., 2012; Carlson et al., 2015; Nawrocki et al., 2015; Bhattacharjee et al., 2016). Although an attractive hypothesis is that the distinct properties of each strain are genotypic and reflect the GI environment of the primary host in which each strain evolved, there are likely many variable environmental factors that contribute to the physical composition of the spore, such as the prominence of different spore proteins and the number of germinant receptors.

Further studies are required to determine which features of the *C. difficile* spore coat and cortex confer resistance to various chemicals and environmental insults. Resistances often arise sequentially, not simultaneously, as additional components are built onto the prespore, as seen in *B. subtilis* (Balassa, 1971; Russell, 1990). Determining the order of resistances as *C. difficile* sporulation progresses will provide insight into the biochemical mechanisms of spore formation and may serve as a useful tool for studying late sporulation events.

## Author Contributions

AE, SK, RP, LJ, SA, and SM contributed to the acquisition and analysis of the data. AE drafted the manuscript and AE, SK, RP, LJ, SA, and SM edited and approved the content.

## Conflict of Interest Statement

The authors declare that the research was conducted in the absence of any commercial or financial relationships that could be construed as a potential conflict of interest.
