# Supplementary material for: Chemical and Stress Resistances of Clostridium difficile Spores and Vegetative Cells
Source: Front Microbiol. 2016 Oct 26;7:1698. doi: 10.3389/fmicb.2016.01698 (PMC5080291; doi:10.3389/fmicb.2016.01698)
Supplement: Supplementary file 1 [file Presentation_1.PDF]

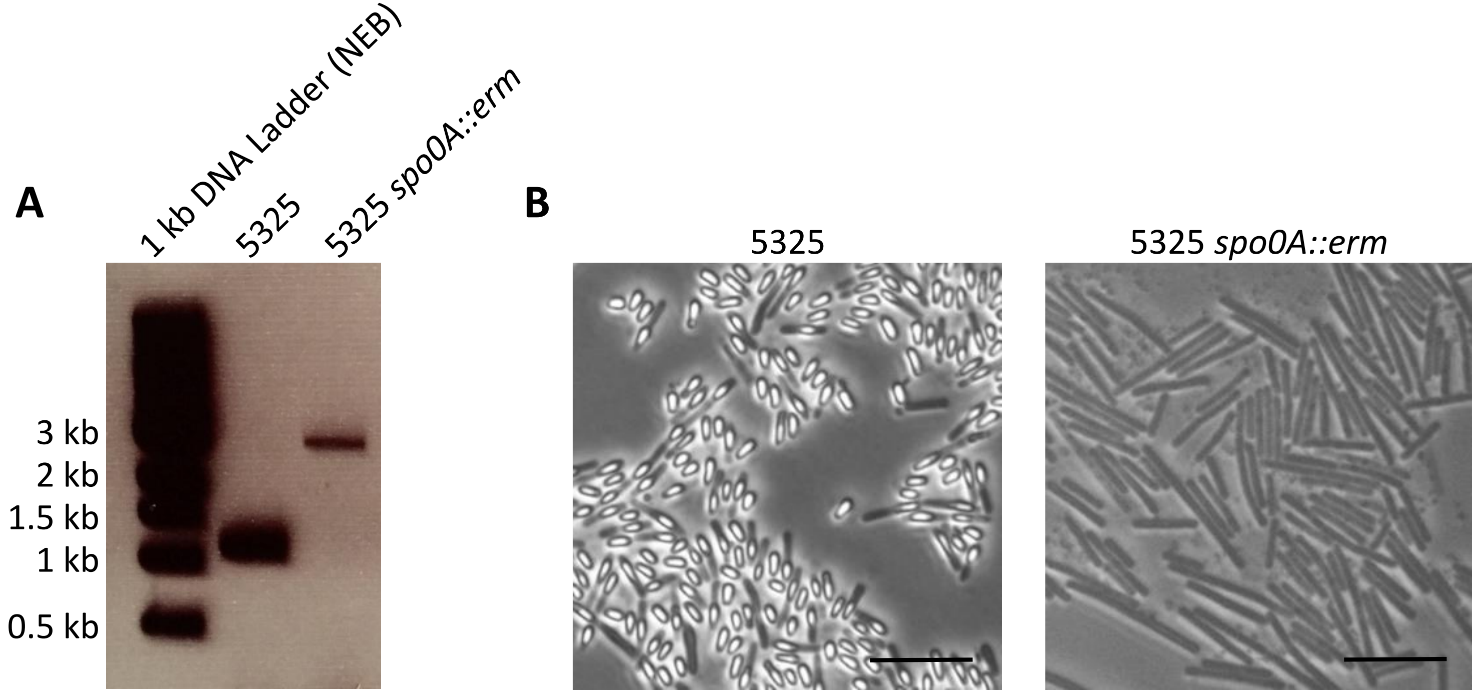

Figure S1. **Confirmation of 5325 *spo0A* mutant.** (**A**) PCR amplification of the *spo0A* locus in strain 5325 and the 5325 *spo0A*::*erm* mutant, demonstrating the insertion of the ~2 kb targetron construct into the *spo0A* gene. (**B**) Phase contrast micrographs of 5325 and 5325 *spo0A*::*erm* on 70:30 sporulation agar at H_{24}. The scale bar represents 10 µm.

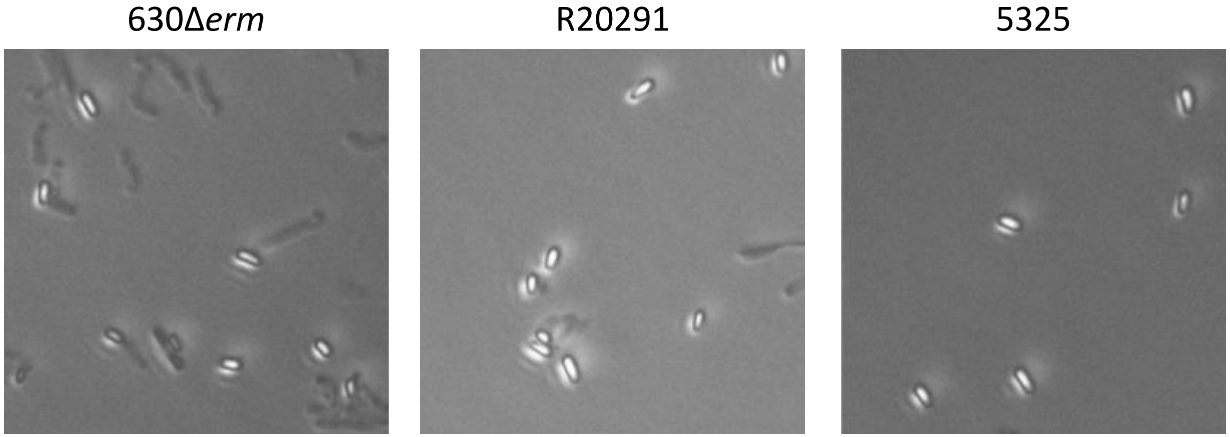

Figure S2. **Isolation of *C. difficile* spores.** Phase contrast micrographs of isolated 630Δ*erm* (012 ribotype), R20291 (027 ribotype) and 5325 (078 ribotype) *C. difficile* spores purified from 70:30 sporulation agar as described in the Materials and Methods.

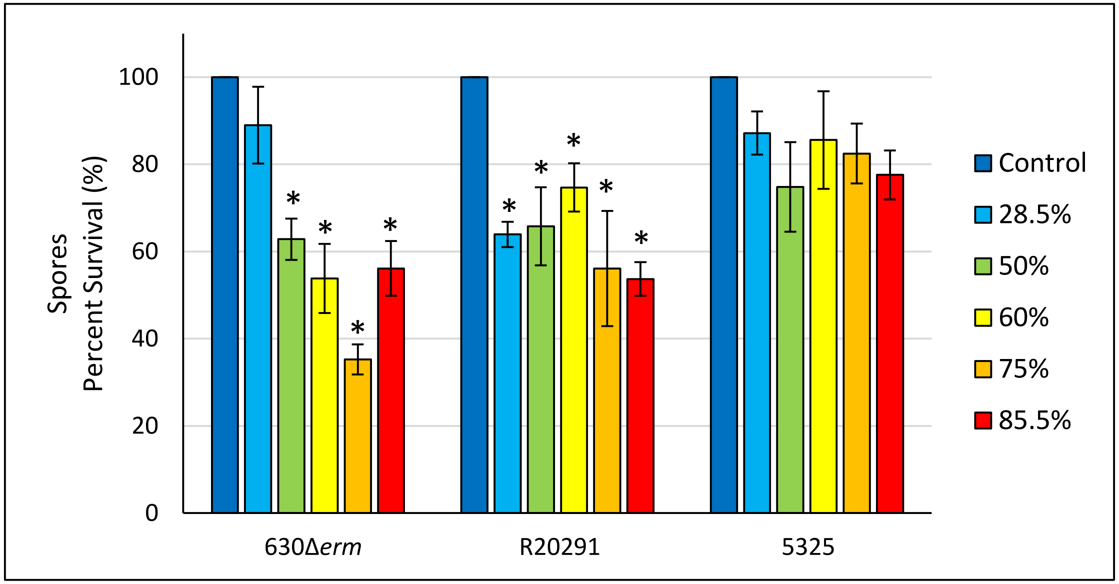

Figure S3. ***C. difficile* spores survive high concentrations of ethanol.** Percent survival (%) of 630Δ*erm*, R20291 and 5325 spores in 1X PBS after exposure to the indicated concentrations (v/v) of ethanol for 15 min (original data is depicted in Fig. 1B). The means and standard error of the means for the percent survival relative to the control condition of three biological replicates are shown. Asterisks indicate a *P* value of <0.05 as determined by two-way ANOVA followed by a Dunnett's multiple comparisons test to compare the condition to the corresponding control of individual strains.

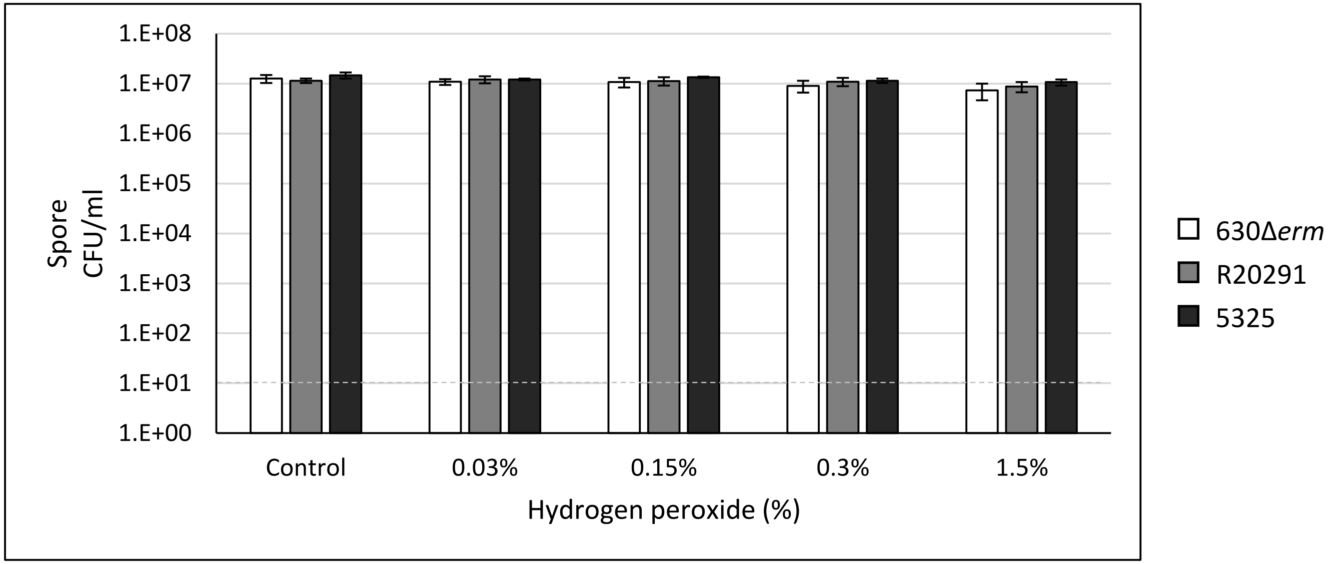

Figure S4. *C. difficile* spores are resistant to hydrogen peroxide. 630Δ*erm*, R20291 and 5325 spores in 1X PBS after a 15 min exposure to the indicated concentration (v/v) of hydrogen peroxide (H_2O_2). The means and standard error of the means for at least three biological replicates are shown; the limit of detection is 10 CFU ml^{-1} and is denoted by a dashed line. Asterisks indicate a *P* value of <0.05 as determined by two-way ANOVA followed by a Dunnett's multiple comparisons test to compare the condition to the corresponding control of individual strains.

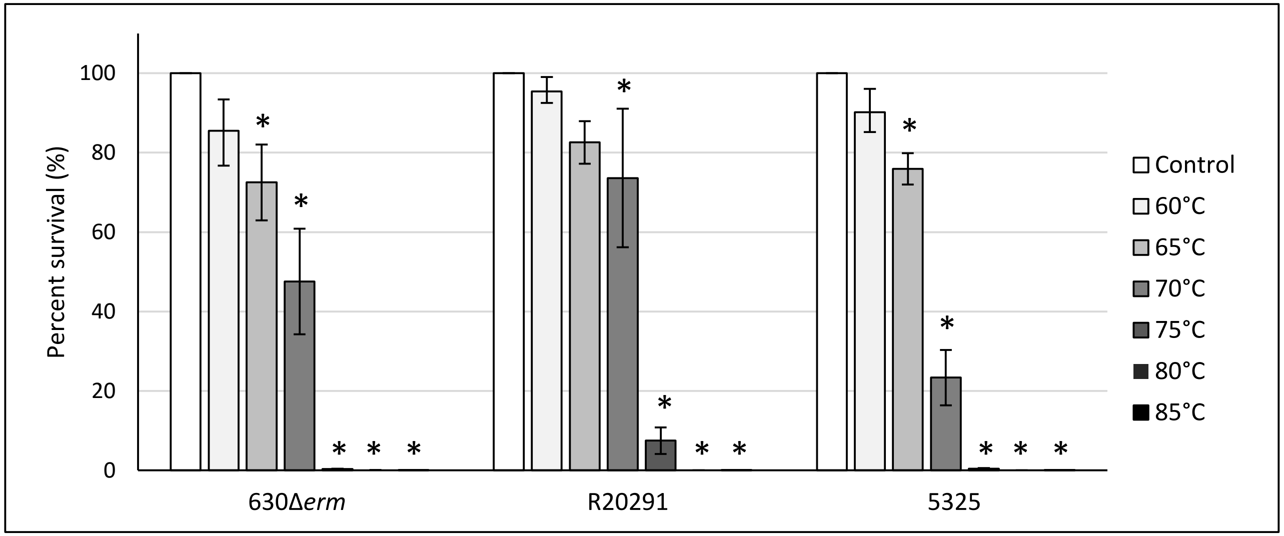

Figure S5. *C. difficile* spores are resistant to high temperatures. Survival of 630Δ*erm*, R20291 and 5325 spores in 1X PBS after a 20 min exposure to the indicated temperature represented as percent survival (%) of total CFU. The means and standard error of the means for at least three biological replicates are shown. Asterisks indicate a *P* value of <0.05 as determined by two-way ANOVA followed by a Dunnett's multiple comparisons test to compare the condition to the corresponding control of individual strains.

Condition tested	630Δ*erm* (CFU ml^{-1})	630 *spo0A* (CFU ml^{-1})	Statistical significance
**Control**	$3.73 \times 10^8 \pm 1.21 \times 10^7$	$3.21 \times 10^8 \pm 1.06 \times 10^7$	ns
**0.0003% H_2O_2**	$3.37 \times 10^8 \pm 4.06 \times 10^6$	$3.10 \times 10^8 \pm 1.70 \times 10^7$	ns
**0.0015% H_2O_2**	$4.01 \times 10^6 \pm 1.26 \times 10^6$	$1.23 \times 10^8 \pm 5.49 \times 10^7$	ns
**0.003% H_2O_2**	$1.67 \times 10^2 \pm 1.07 \times 10^2$	$8.03 \times 10^2 \pm 7.93 \times 10^2$	ns
**9.5% ethanol**	$3.42 \times 10^8 \pm 3.21 \times 10^7$	$2.00 \times 10^8 \pm 1.03 \times 10^8$	ns
**14.25% ethanol**	$8.41 \times 10^7 \pm 6.17 \times 10^7$	$1.35 \times 10^8 \pm 9.73 \times 10^6$	ns
**19% ethanol**	$4.60 \times 10^2 \pm 2.32 \times 10^2$	$9.45 \times 10^3 \pm 9.43 \times 10^3$	ns
**O_2 (3 h)**	$4.67 \times 10^7 \pm 5.78 \times 10^6$	$1.44 \times 10^8 \pm 5.14 \times 10^7$	ns
**400 mg/L FC**	$1.68 \times 10^3 \pm 1.36 \times 10^3$	$3.54 \times 10^7 \pm 3.53 \times 10^7$	ns
**1000 mg/L FC**	$1.64 \times 10^3 \pm 1.55 \times 10^3$	$1.00 \times 10^1 \pm 1.00 \times 10^1$	ns

Figure S6. The isogenic parent, 630Δ*erm*, exhibits similar levels of resistance to stressors as its *spo0A* derivative (630 *spo0A*). The average number of viable CFU per ml recovered on BHIS + 0.1% taurocholate plates for each strain after exposure to the indicated condition, as described in the Materials and Methods. The means and standard error of the means for at least three biological replicates are shown. The statistical significance comparing the 630Δ*erm* parent and the 630 *spo0A* strains for each condition was determined using a two-way ANOVA followed by a Tukey's multiple comparisons test (ns, not significant). FC, free chlorine.
